# Long-term brain volume trajectories and lifestyle associations in cognitively normal adults: the BRAIN-STRIDE study

**DOI:** 10.1093/braincomms/fcag343

**Published:** 2026-09-07

**Authors:** Shohei Fujita, Susumu Mori, Kengo Onda, Shouhei Hanaoka, Yukihiro Nomura, Takahiro Nakao, Akifumi Hagiwara, Yasuhiro Hagiwara, Hidemasa Takao, Takeharu Yoshikawa, Osamu Abe

**Affiliations:** Department of Radiology, The University of Tokyo, Tokyo, 113-8655, Japan; Department of Radiology, Juntendo University, Tokyo, 113-8421, Japan; Athinoula A. Martinos Center for Biomedical Imaging, Massachusetts General Hospital, Boston, MA 02129, USA; Department of Radiology, Harvard Medical School, Boston, MA 02115, USA; Russell H. Morgan Department of Radiology and Radiological Science, Johns Hopkins University School of Medicine, Baltimore, MD 21287, USA; F.M. Kirby Research Center for Functional Brain Imaging, Kennedy Krieger Institute, Baltimore, MD 21205, USA; Russell H. Morgan Department of Radiology and Radiological Science, Johns Hopkins University School of Medicine, Baltimore, MD 21287, USA; Department of Radiology, The University of Tokyo, Tokyo, 113-8655, Japan; Department of Computational Diagnostic Radiology and Preventive Medicine, The University of Tokyo Hospital, Tokyo, 113-8655, Japan; Center for Frontier Medical Engineering, Chiba University, Chiba, 263-8522, Japan; Department of Computational Diagnostic Radiology and Preventive Medicine, The University of Tokyo Hospital, Tokyo, 113-8655, Japan; Department of Radiology, The University of Tokyo, Tokyo, 113-8655, Japan; Department of Radiology, Juntendo University, Tokyo, 113-8421, Japan; Department of Biostatistics, School of Public Health, The University of Tokyo, Tokyo, 113-0033, Japan; Department of Radiology, The University of Tokyo, Tokyo, 113-8655, Japan; Department of Computational Diagnostic Radiology and Preventive Medicine, The University of Tokyo Hospital, Tokyo, 113-8655, Japan; Department of Radiology, The University of Tokyo, Tokyo, 113-8655, Japan

**Keywords:** ageing, longitudinal studies, lifestyle, brain volume, cognition

## Abstract

Brain ageing is a major determinant of cognitive health, yet the extent of inter-individual variability in structural trajectories and its links to lifestyle factors remain incompletely understood. We conducted a single-centre, prospective longitudinal cohort study within an urban academic health screening programme in Japan (November 2006 to April 2021), with up to 15 years of follow-up. Among 10 209 individuals who underwent screening, those with more than 10 years of serial visits were eligible; 186 participants were excluded for major intracranial findings, 3 for cognitive decline during follow-up and 3 for contraindications or ineligibility, yielding 653 cognitively normal adults [207 women (31.6%); mean (SD) age at baseline, 55.1 (9.3) years]. Participants underwent serial magnetic resonance imaging and Mini-Mental State Examination, along with baseline assessments of lifestyle and vascular risk factors including body mass index, blood pressure, smoking exposure and lipid profile. Annual percentage change in whole-brain and regional volumes was estimated using linear mixed-effects models after harmonization for scanner effects, and participants were categorized as rapid atrophy, typical, or atrophy-resistant based on percentile thresholds. Across 7915 MRI scans, whole-brain volume decline accelerated from 0.24% per year in the 40s to 0.44% per year in the 70s. Individuals classified as having rapid atrophy in their 40s exhibited rates of decline of 0.46% per year, comparable to population averages in the 70s, whereas atrophy-resistant individuals in their 70s demonstrated rates as low as 0.27% per year. Faster whole-brain atrophy was associated with higher body mass index [*β* = 0.027; 95% confidence interval (CI), 0.0058–0.049], greater smoking exposure (*β* = 0.032; 95% CI, 0.012–0.053), elevated diastolic blood pressure (*β* = 0.044; 95% CI, 0.021–0.067) and higher low-density lipoprotein cholesterol (*β* = 0.043; 95% CI, 0.021–0.065) (all *P* < 0.01). Utilizing an uncommonly long and densely sampled longitudinal design, this study demonstrates that structural brain ageing among cognitively normal adults follows highly heterogeneous trajectories, spanning from marked decline to near-complete resistance. Modifiable lifestyle and vascular risk factors were associated with faster atrophy, supporting opportunities for risk stratification and targeted prevention strategies.

## Teaser

Fujita *et al.* report that serial MRI up to 15 years reveals marked individual differences in brain volume loss among cognitively normal adults, including rapid-atrophy and atrophy-resistant phenotypes. Faster whole-brain loss is linked to obesity, smoking history, higher diastolic blood pressure and adverse cholesterol profiles, supporting MRI-based risk stratification.

## Graphical Abstract

**Graphical Abstract:**
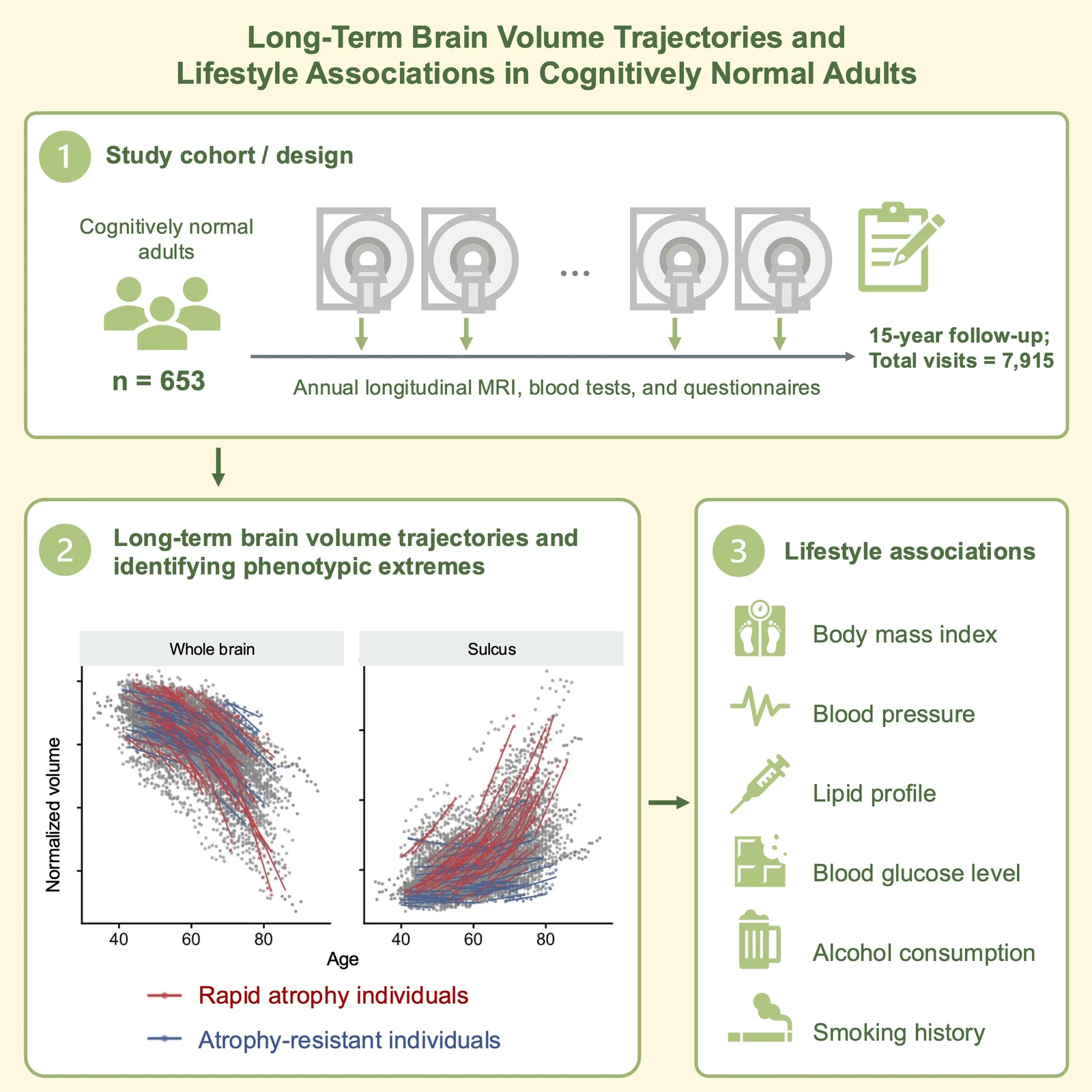

## Introduction

Age-related brain atrophy has long been regarded as a fundamental and largely inevitable biological hallmark of human ageing.^1^ Our current understanding of age-related brain changes is based primarily on population-based cross-sectional studies^2-4^ and a limited number of longitudinal studies.^5-8^ Recent longitudinal investigations have revealed that (i) the individual trajectory underlying brain ageing based on population data is challenging to discern through cross-sectional analysis; (ii) discrepancies exist in age-related trends in population- and individual-level data; and (iii) there appears to be a subset of individuals who deviate considerably from the population average.^9-11^

The variations in age-related changes among individuals are not yet fully understood and may reflect differences in underlying biology, lifestyle, or other modifiable factors.^12^ The term ‘SuperAger’ has been used in previous studies to describe older adults with preserved brain structure and cognition,^13,14^ but this represents only one end of a broader spectrum. At the other end, a small subset of otherwise healthy adults with normal cognition may have brain volume loss at rates comparable to those observed in clinical populations.^10^ Capturing both atrophy-resistant and rapid-atrophy phenotypes contributes to a better understanding of brain ageing. However, most studies have focused on group-level trends, often obscuring individual variation. Cross-sectional designs are limited in detecting dynamic intra-individual changes,^9^ and even longitudinal studies often lack the duration or sample size to capture meaningful divergence. This is especially true in younger adults, for whom data are scarce.^2^

In this study, we leveraged a large-scale longitudinal MRI dataset spanning over 15 years with annual brain MRI examinations as part of a health screening programme. This study aimed to quantify variability in brain atrophy rates and identify individuals at both extremes of the distribution. To our knowledge, the combination of annual acquisition cadence, multi-decade follow-up and sample size is rare among longitudinal neuroimaging cohorts, enabling detection of subtle intra-individual trajectories that shorter or sparser designs may not resolve. We characterized the volume trajectories of atrophy-resistant and rapid-atrophy groups and explored the associated lifestyle factors. Our results highlight structural heterogeneity in normal ageing and may inform future strategies for risk stratification and prevention.

## Materials and methods

### Study design and participants

The Study of TRajectories and Inter-individual Differences in Early to late adulthood (BRAIN-STRIDE) study is a long-term longitudinal prospective cohort study conducted in Japan at a single urban academic university hospital. The present study is a secondary analysis of the cohort previously reported.^10^ While the prior publication focused on descriptive analyses of age-binned group averages, the current analysis characterizes rapid-atrophy and atrophy-resistant phenotypes and examines associations with lifestyle factors, which were not addressed previously. The study was approved by the Institutional Review Board of the University of Tokyo Hospital. Written informed consent was obtained from all participants.

The study population consisted of individuals enrolled in a comprehensive health screening programme that included annual high-resolution brain MRI examinations between November 2006 and April 2021. Participants were eligible for inclusion if they underwent at least 10 annual scans during this period and showed no clinical evidence of cognitive impairment. Clinical history was documented at each visit, and any indication of cognitive impairment was evaluated by an internal medicine physician. The assessment followed a structured, multi-step process in which participants first completed a self-administered questionnaire. This was then reviewed by a nurse and subsequently verified by the physician during a clinical examination. Cognitive function was assessed using the Mini-Mental State Examination (MMSE)^15^ and the Wechsler Memory Scale-Revised test.^16^ Participants were excluded if they had an MMSE score of ≤24, a history of neurological or psychiatric disorders, or major intracranial abnormalities such as haemorrhage, infarction, or tumours on MRI. Because the aim of the study was to quantify extremely subtle longitudinal changes in brain volume, even minor structural abnormalities (e.g. small lacunar infarcts, solitary microbleeds, or arachnoid cysts) were considered potential sources of confounding and were therefore excluded.

### MRI acquisition

All participants underwent annual brain MRI examinations using a 3-T system as part of the institutional health screening programme. To ensure consistency in data acquisition, the imaging protocol was harmonized throughout the study period, despite hardware and software upgrades. To further account for these upgrades, multivariate linear regression modelling was applied, as described later. From November 2006 to December 2017, imaging was performed using a Signa EXCITE and a Discovery MR750 scanner (GE HealthCare), with a fast spoiled gradient-echo sequence (repetition time: 6.4 ms; echo time: 2.0 ms; inversion time: 450 ms; flip angle: 15°; matrix size: 256 × 256; field of view: 25 cm; voxel size: 0.98 × 0.98 × 1.0 mm).^17^ From January 2018 to April 2021, imaging was performed using a Biograph mMR scanner (Siemens Healthineers) with a magnetization-prepared rapid gradient-echo sequence (repetition time: 1660 ms; echo time: 2.4 ms; inversion time: 910 ms; flip angle: 8°; same matrix and field of view; voxel size: 0.98 × 0.98 × 1.0 mm).^18^

All images were acquired using either an 8- or 16-channel head coil. Axial T2-weighted, fluid-attenuated inversion recovery and diffusion-weighted images were obtained for screening, in addition to T1-weighted imaging. Image quality was inspected on-site by MRI technologists, and all scans were independently reviewed by two radiologists to screen for structural abnormalities and clinically relevant lesions.

### Brain volume measurement and longitudinal harmonization

All T1-weighted images were processed using an automated multi-atlas segmentation pipeline (MVision, CorporateM, Tokyo, Japan), which is based on the MriCloud framework^19^ developed at Johns Hopkins University. This pipeline parcellates the entire brain into predefined anatomical regions of interest and calculates the respective volumes.^19,20^ Image processing was performed on a stand-alone Linux workstation by a trained technician who was blinded to the clinical and demographic data of the participants.

To account for potential confounding effects of scanner upgrades and protocol changes over the study period, we harmonized all regional measures using longitudinal ComBat.^21^ This method extends the original ComBat empirical-Bayes framework,^22^ which estimates and removes additive and multiplicative batch effects, by incorporating a linear mixed-effects model that explicitly accounts for within-subject correlation across repeated measurements. After estimation, scanner effects were removed while covariate terms and random intercepts were re-applied, yielding harmonized values that preserve individual longitudinal trajectories. Harmonization was performed separately for each metric.

After harmonization of scanner effects, absolute volumes were normalized by calculating the ratio of each structure’s volume at each time point to its baseline volume, yielding relative volume measures. Global brain structures including the whole brain, cortical grey matter, white matter, sulci and ventricles were assessed. To evaluate regional variation, the frontal, occipital, parietal and temporal lobes, as well as the hippocampus, were also examined.

### Identification of brain atrophy subtypes in cognitively normal adults

Longitudinally harmonized brain region volumes were normalized to each participant’s baseline scan, such that all trajectories were anchored at 1.0 at time zero. To estimate individual atrophy rates, we applied linear mixed-effects models with follow-up time as the sole longitudinal predictor. The model specification included a fixed intercept set to 1, no random intercept (since all subjects were aligned at baseline) and a random slope for follow-up time to capture individual-specific atrophy trajectories. Main effects were not included, as baseline-normalized modelling focuses exclusively on longitudinal change rather than cross-sectional differences. Instead, interaction terms between follow-up time and baseline age and sex were included to assess their associations with the rate of regional volume change. The resulting individual slopes represent annual percentage change from baseline and were extracted for each brain region for subsequent analysis.

To investigate individual variability in brain ageing trajectories, participants were classified into atrophy subgroups based on longitudinal changes in brain structure. Within every combination of region and age decade, the 10th and 90th percentiles of the slope distribution defined rapid-atrophy group, typical group and atrophy-resistant group, respectively. Stability of these labels was evaluated with 2000 bootstrap resamples drawn with replacement while preserving the region by decade structure. Each resample repeated the percentile based labelling and stored the median slope difference between resistant and rapid groups. For every participant, the proportion of resamples carrying the labels rapid or resistant served as the empirical probability of that classification. A final label was assigned when either probability reached at least 80%; otherwise the participant remained in the typical group. To identify distinct regional atrophy patterns characterizing the rapid-atrophy and atrophy-resistant groups, subject-specific annual atrophy rates were visualized and compared descriptively between the groups. These 10th/90th thresholds were selected to capture the tails of the distribution while retaining a sufficient number of participants within each decade for stable estimation. To evaluate the robustness of this choice, a sensitivity analysis using alternative percentile thresholds (5th/95th and 15th/85th), with group definitions otherwise unchanged, was also performed.

To assess whether individuals with extreme whole-brain atrophy also exhibited extreme tissue-specific atrophy, we performed a rank-based analysis. For each participant, percentile ranks were computed across the cohort for whole-brain atrophy, cortical atrophy, white matter atrophy, ventricular enlargement and sulcal expansion. Participants in the top and bottom deciles (≥90th and ≤10th percentile) of whole-brain atrophy were identified, and their tissue-specific percentile ranks were displayed using participant-level heatmaps. The proportion of participants in these whole-brain extremes who also fell within the corresponding tissue-specific deciles was then quantified to characterize the degree of overlap.

### Associations with lifestyle and clinical factors

To examine associations between lifestyle factors and longitudinal brain atrophy, we constructed linear mixed-effects models with individual-level random slopes. Baseline lifestyle variables were dichotomized based on standard clinical thresholds to account for potential non-linear effects and to improve interpretability: body mass index (BMI) ≥25 kg/m^2^, diastolic blood pressure ≥80 mmHg, systolic blood pressure ≥140 mmHg, any smoking exposure, fasting glucose ≥126 mg/dl, low-density lipoprotein (LDL)-cholesterol ≥130 mg/dl and high-density lipoprotein (HDL)-cholesterol <40 mg/dl. These binary flags were entered into the linear mixed-effects model only as interaction terms with follow-up time to estimate their association with longitudinal changes in normalized brain volume. Since all brain volumes were baseline-normalized, main effects were not included. Fixed effects also included baseline age and sex, and random slopes were modelled at the individual level. The interaction term coefficients (*β*) represent the difference in annual whole-brain atrophy rate between participants who exceed a clinical threshold and those who do not. Time-varying modelling was avoided due to limited expected impact on atrophy rates and increased model complexity.

### Statistical analysis

All analyses were performed using R (version 4.4.3, R Core Team, Vienna, Austria) with the tidyverse package,^23^ lme4 package,^24^ and broom.mixed package.^25^ Statistical significance was set at two-sided *P*-values of <0.05.

## Results

### Participant characteristics

**Figure 1:**
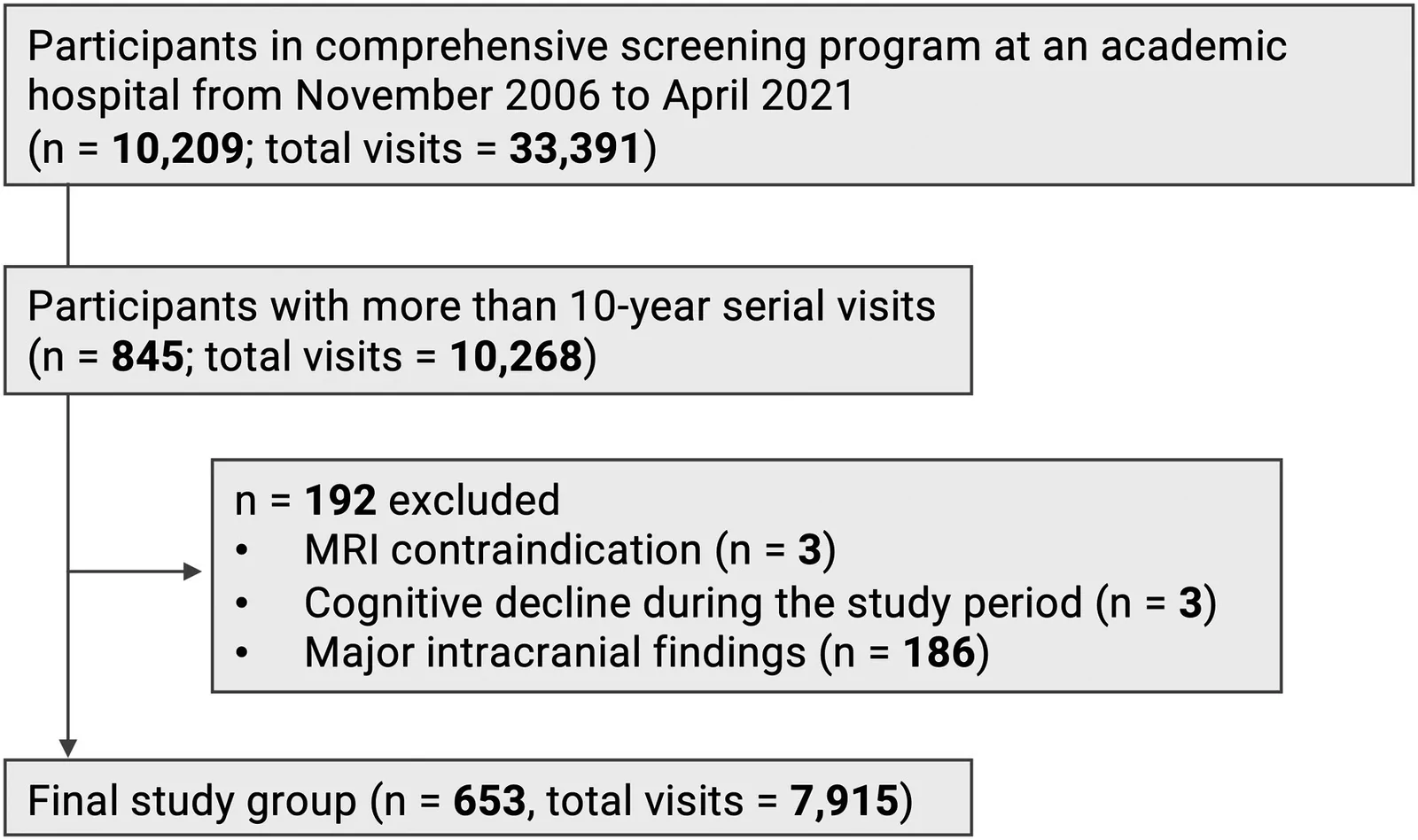
**Participant inclusion and exclusion flowchart**.

A total of 10 209 individuals (33 391 visits) were screened. Of these, 845 met the criterion of having at least 10 scans. After excluding three participants with MRI contraindication and three participants with cognitive impairment during the study period and 186 participants with major intracranial pathologies, 653 adults with normal cognition {206 women, median age: 55 years [interquartile range (IQR): 47–62] at baseline} were included in the final analysis (Fig. 1). These participants were followed longitudinally for up to 15 years, with a mean (SD) follow-up duration of 11.5 (1.8) years, and a total of 7915 MRI scans were analysed. The baseline clinical and demographic characteristics are summarized in Table 1. All participants completed at least 10 serial MRI examinations as part of a health screening protocol.

**Table 1:** Characteristics of the participants

Characteristics	Data (unique individuals = 653)
Age at baseline, mean [SD], median (IQR)	55.1 [9.3], 55 (47–62)
Follow-up period, mean [SD], median (IQR)	11.5 [1.8], 11.5 (10.2–13.1)
Sex	
Women, *n* (%)	206 (32)
Men, *n* (%)	447 (68)
Mini-Mental State Examination, mean [SD], median (IQR)	29.4 [0.9], 30 (29–30)
Wechsler Memory Scale-Revised, mean [SD], median (IQR)	33.8 [6.5], 34 (30–38)

Note: Categorical data are reported as numerators, and the corresponding percentages are in parentheses.

### Data harmonization

Mixed-effects modelling showed that, before harmonization, scanner (‘batch’) accounted for 7.6–14.9% of the variance in 8 of 10 brain regions evaluated and was statistically significant in Kenward–Roger tests (*P* < 1 × 10^−16^ for most regions; Supplementary Table 1). The ventricle showed no measurable additive batch effect (0%, *P* = 0.156).

After applying longitudinal ComBat, batch-related variance fell to ≤0.1% in every region, and the batch term became non-significant for all regions (*P* = 0.536–0.999). Supplementary Figure 1 presents box plots for inter-protocol offsets. Intra-class correlation coefficients remained high and virtually unchanged (0.90–0.98 before versus 0.90–0.98 after), indicating that true within-subject trajectories were preserved.

### Group level brain volume changes

**Figure 2:**
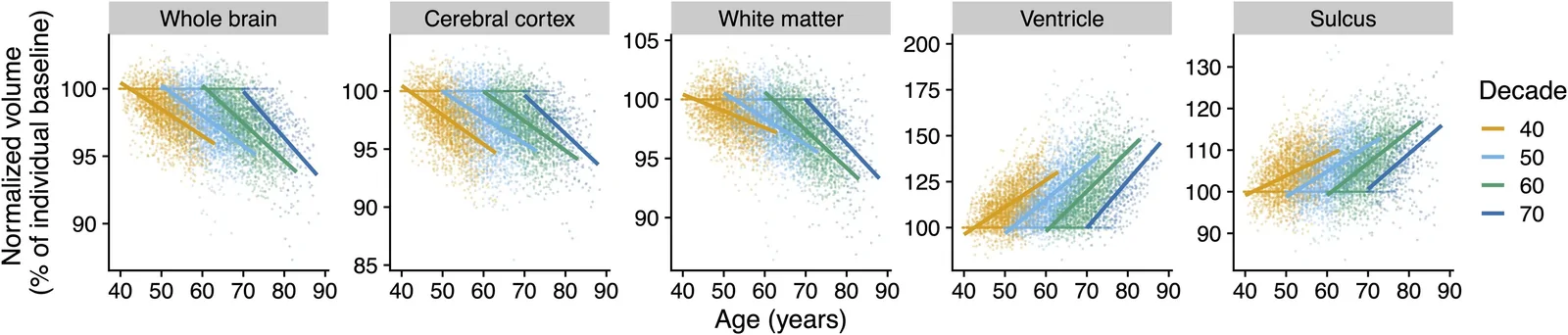
**Decade-specific trajectories of global brain compartments**. Each panel plots subject-wise volumes normalized to individual baseline (vertical axis) against age at scan (horizontal axis). Points are colour-coded by the decade in which participants entered the study; solid lines depict separate decade-wise ordinary least-squares linear fits. These fits are presented for descriptive visualization only; no formal hypothesis testing or *post hoc* comparisons were performed. Each point represents a normalized structure volume from a single MRI exam. The numbers of MRI examinations in each age decade were as follows: 1269 in the 40s, 2624 in the 50s, 2553 in the 60s and 1256 in the 70s. The numbers of participants in each age decade at study entry were 216, 214, 174 and 26, respectively.

Figure 2 illustrates decade-stratified trajectories for global brain compartments. Whole-brain, cerebral-cortex and white-matter volumes show linear declines with age, whereas ventricular and sulcal volumes expand reciprocally. The slopes for white-matter decline accelerate with advancing age, as evidenced by progressively steeper negative slopes in participants who entered the study in their 60s and 70s.

**Figure 3:**
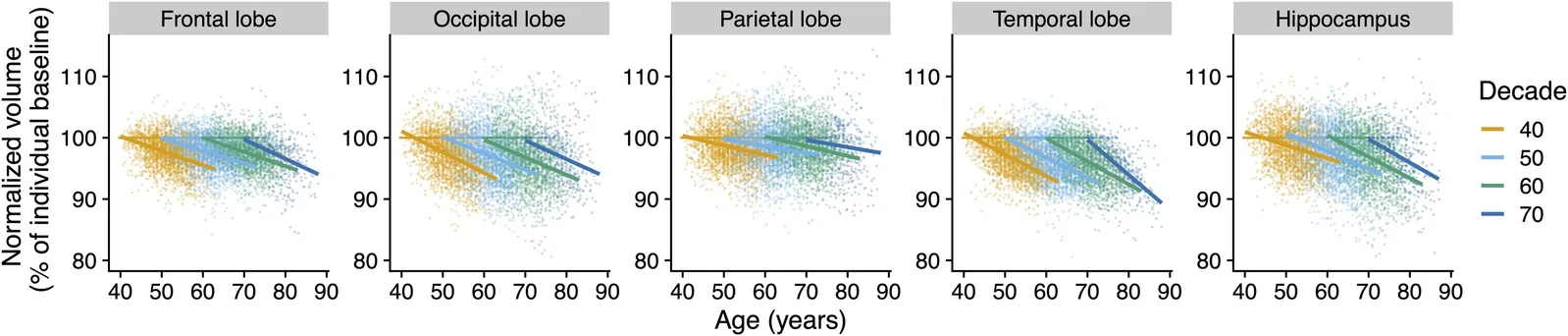
**Decade-specific trajectories of brain region volumes**. Each panel plots subject-wise volumes normalized to individual baseline (vertical axis) against age at scan (horizontal axis). Points are colour-coded by the decade in which participants entered the study; solid lines depict separate decade-wise ordinary least-squares linear fits. These fits are presented for descriptive visualization only; no formal hypothesis testing or *post hoc* comparisons were performed. Brain regions including frontal, occipital and parietal lobes and hippocampus are shown. Each point represents a normalized structure volume from a single MRI exam. The numbers of MRI examinations in each age decade were as follows: 1269 in the 40s, 2624 in the 50s, 2553 in the 60s and 1256 in the 70s. The numbers of participants in each age decade at study entry were 216, 214, 174 and 26, respectively.

Figure 3 illustrates decade-stratified trajectories for lobar regions. Frontal, occipital and parietal cortices demonstrate similar decade-wise slopes, suggesting uniform atrophy across mid-to-late adulthood. By comparison, the temporal cortex and hippocampus exhibit pronounced acceleration.

### Atrophy group classification and longitudinal brain volume changes

**Figure 4:**
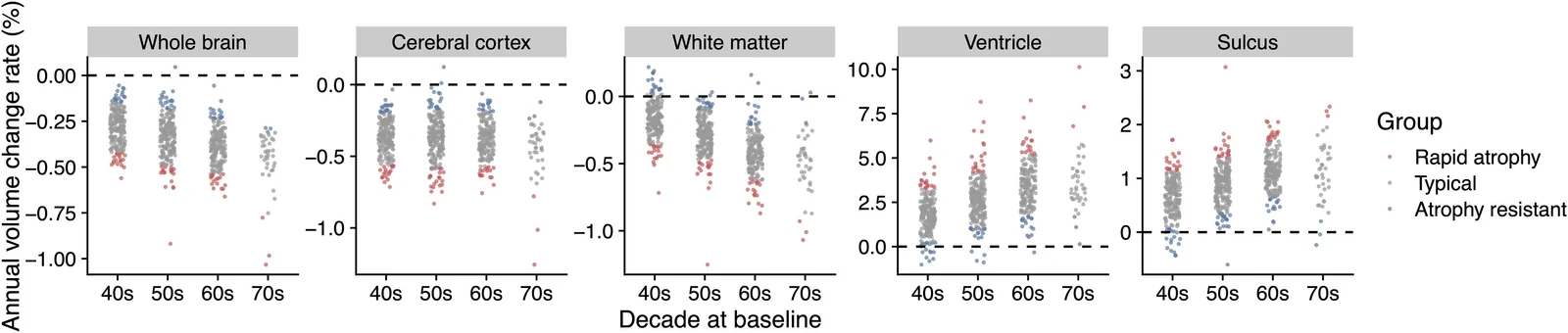
**Distribution of individual atrophy rates across age decades**. Each dot represents the annual percentage change in volume for a single participant, colour-coded by data-driven phenotype (rapid, typical, or resistant). Results are displayed separately for each age decade (40s, *n* = 205; 50s, *n* = 217; 60s, *n* = 186; and 70s, *n* = 40) and for five major brain compartments. Negative values denote tissue loss, whereas positive values in ventricles and sulci indicate expansion of cerebrospinal-fluid spaces. Rapid subjects cluster below the 10th-percentile threshold, resistant subjects above the 19th-percentile threshold, and typical subjects occupy the intermediate range, illustrating the heterogeneity of brain-wide atrophy even within the same age band. The figure is presented for descriptive visualization only; no formal hypothesis testing or *post hoc* comparisons were performed.

Longitudinal mixed-effects analysis revealed substantial inter-individual variability in age-related brain changes. Figure 4 displays the annual slopes, colour-coded by phenotype and arranged by decade. The spread of slopes widens from the 40s to the 70s, indicating that divergence in brain ageing becomes more pronounced with advancing age. In the typical group, whole-brain and cerebral-cortex slopes cluster around −0.4% per year, whereas the gap between rapid and resistant phenotypes is greatest for white-matter loss and ventricular expansion. A complete summary of atrophy rates by group and age decade is provided in Table 2. Notably, atrophy-resistant individuals demonstrated longitudinal changes close to zero or even slight volume increases over more than a decade up to their 70s. In contrast, the rapid-atrophy group exhibited a markedly high rate of atrophy. For example, the whole-brain atrophy rate in participants in the 40s (0.46%/year) was comparable to the population-level atrophy rate typically observed in the 70s (0.44%/year). Conversely, the atrophy-resistant group in their 70s (0.27%/year) exhibited atrophy rates similar to the population-level rates observed in individuals in their 40s (0.27%/year). The qualitative conclusions were preserved across threshold definitions. The widening of inter-individual variability with age remained evident, and the rapid-atrophy and atrophy-resistant groups remained well separated in their slope distributions under each definition. Full results of the sensitivity analysis are provided in Supplementary Table 2.

**Table 2:** Decade-specific annual volume change across global compartments

Structure	Decade of baseline age	Typical group annual % (IQR)	Rapid-atrophy group annual % (IQR)	Atrophy-resistant group annual % (IQR)
Whole brain	40s	−0.27 (−0.34 to −0.22)	−0.46 (−0.48 to −0.44)	−0.11 (−0.13 to −0.08)
	50s	−0.33 (−0.40 to −0.28)	−0.52 (−0.53 to −0.51)	−0.15 (−0.18 to −0.12)
	60s	−0.40 (−0.46 to −0.35)	−0.58 (−0.62 to −0.55)	−0.21 (−0.23 to −0.19)
	70s	−0.44 (−0.50 to −0.35)	−0.76 (−0.93 to −0.74)	−0.27 (−0.29 to −0.24)
Cerebral cortex	40s	−0.37 (−0.42 to −0.31)	−0.61 (−0.65 to −0.58)	−0.13 (−0.16 to −0.08)
	50s	−0.37 (−0.44 to −0.30)	−0.63 (−0.67 to −0.60)	−0.13 (−0.17 to −0.10)
	60s	−0.38 (−0.46 to −0.32)	−0.63 (−0.65 to −0.59)	−0.14 (−0.17 to −0.11)
	70s	−0.38 (−0.48 to −0.34)	−0.73 (−0.96 to −0.69)	−0.21 (−0.24 to −0.19)
White matter	40s	−0.16 (−0.23 to −0.09)	−0.40 (−0.44 to −0.36)	−0.05 (−0.07 to −0.03)
	50s	−0.28 (−0.37 to −0.22)	−0.53 (−0.57 to −0.49)	−0.06 (−0.08 to −0.04)
	60s	−0.43 (−0.51 to −0.36)	−0.70 (−0.74 to −0.65)	−0.16 (−0.19 to −0.09)
	70s	−0.51 (−0.62 to −0.41)	−0.89 (−0.99 to −0.86)	−0.19 (−0.21 to −0.06)
Ventricle	40s	1.85 (1.33 to 2.35)	−0.07 (−0.47 to 0.08)	3.68 (3.49 to 4.76)
	50s	2.53 (1.91 to 3.08)	0.83 (0.43 to 0.96)	5.10 (4.61 to 5.83)
	60s	3.23 (2.47 to 3.86)	1.37 (1.02 to 1.46)	6.03 (5.59 to 7.05)
	70s	3.23 (2.67 to 3.29)	2.11 (0.81 to 1.39)	7.38 (6.88 to 8.03)
Sulcus	40s	0.66 (0.45 to 0.86)	1.14 (0.27 to 2.00)	1.26 (1.17 to 1.36)
	50s	0.88 (0.66 to 1.05)	0.24 (0.12 to 0.30)	1.49 (1.41 to 1.61)
	60s	1.10 (0.94 to 1.28)	0.48 (0.42 to 0.56)	1.78 (1.74 to 1.84)
	70s	1.15 (0.94 to 1.49)	0.22 (0.01 to 0.33)	2.17 (2.10 to 2.25)

Note: Values are median annual percentage change with first and third quartiles in parentheses. WM, white matter; IQR, interquartile range.

**Figure 5:**
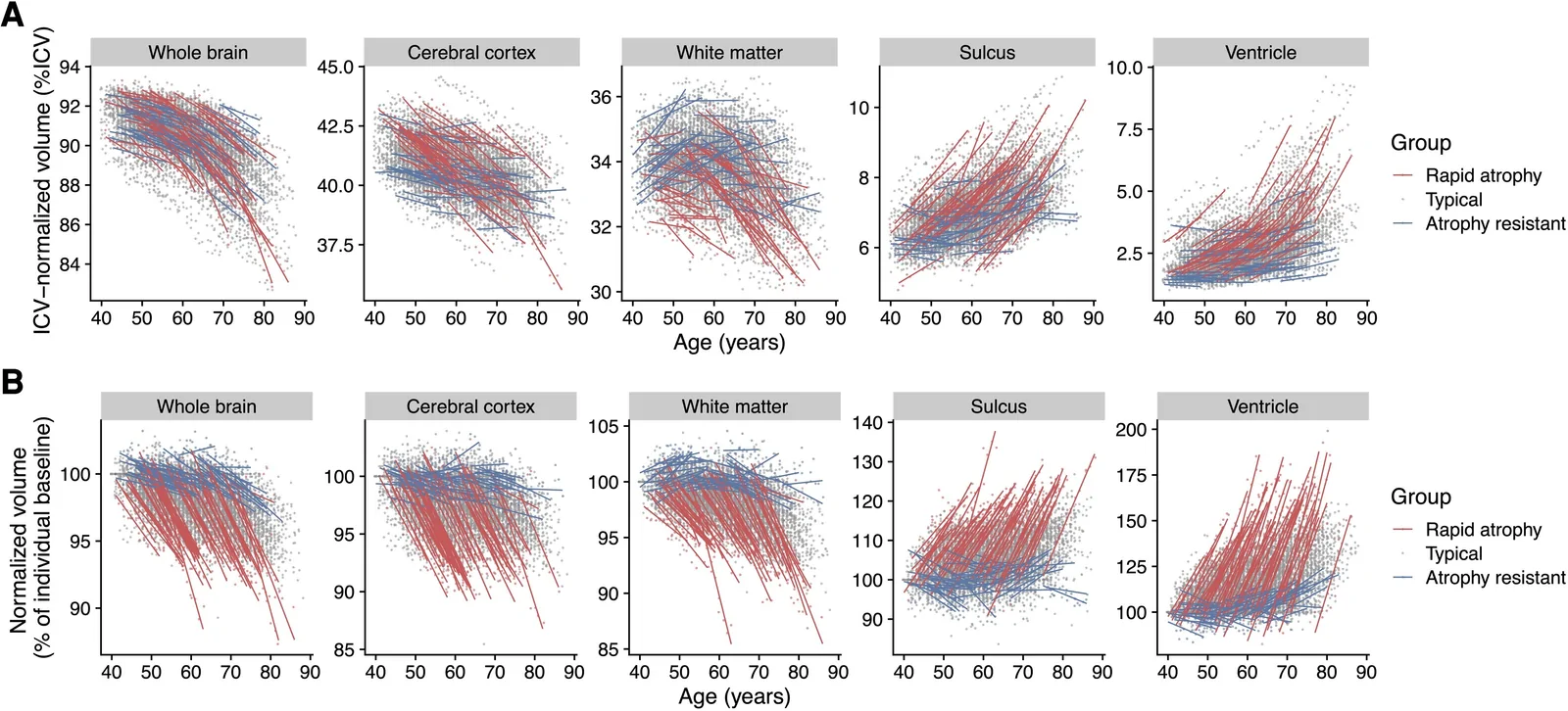
**Age–volume trajectories by phenotypic group**. (**A**) Volume expressed as a percentage of intracranial volume (% ICV) and (**B**) volume normalized to individual baseline. Scatterplots of baseline-normalized volume versus age are overlaid with subject-specific ordinary least-squares linear fits, distinguished by phenotype. Rapid individuals follow steeper downwards paths in tissue compartments and flatter or ascending paths in fluid spaces, whereas resistant individuals show the opposite pattern, with typical participants distributed between the extremes. The fitted trajectories are presented for descriptive visualization only; no formal hypothesis testing or *post hoc* comparisons were performed. Each point represents a normalized structure volume from a single MRI exam, and each line represents the fitted trajectory for an individual participant. Participants by decade at study entry were: 40s, *n* = 216; 50s, *n* = 214; 60s, *n* = 174; and 70s, *n* = 26.

Figure 5 shows these differences in a longitudinal context by plotting baseline-normalized volumes against age and overlaying within-person regression lines. These findings suggest that variability in age-related brain atrophy is much greater than can be inferred from group-level fitting (Figs. 2 and 3). Specifically, a subset of individuals experiences remarkably high rates of brain atrophy even during midlife without cognitive manifestations (‘silent atrophy’) while a others demonstrate exceptional resilience even into advanced age.

### Brain atrophy subtypes in cognitively normal adults

**Figure 6:**
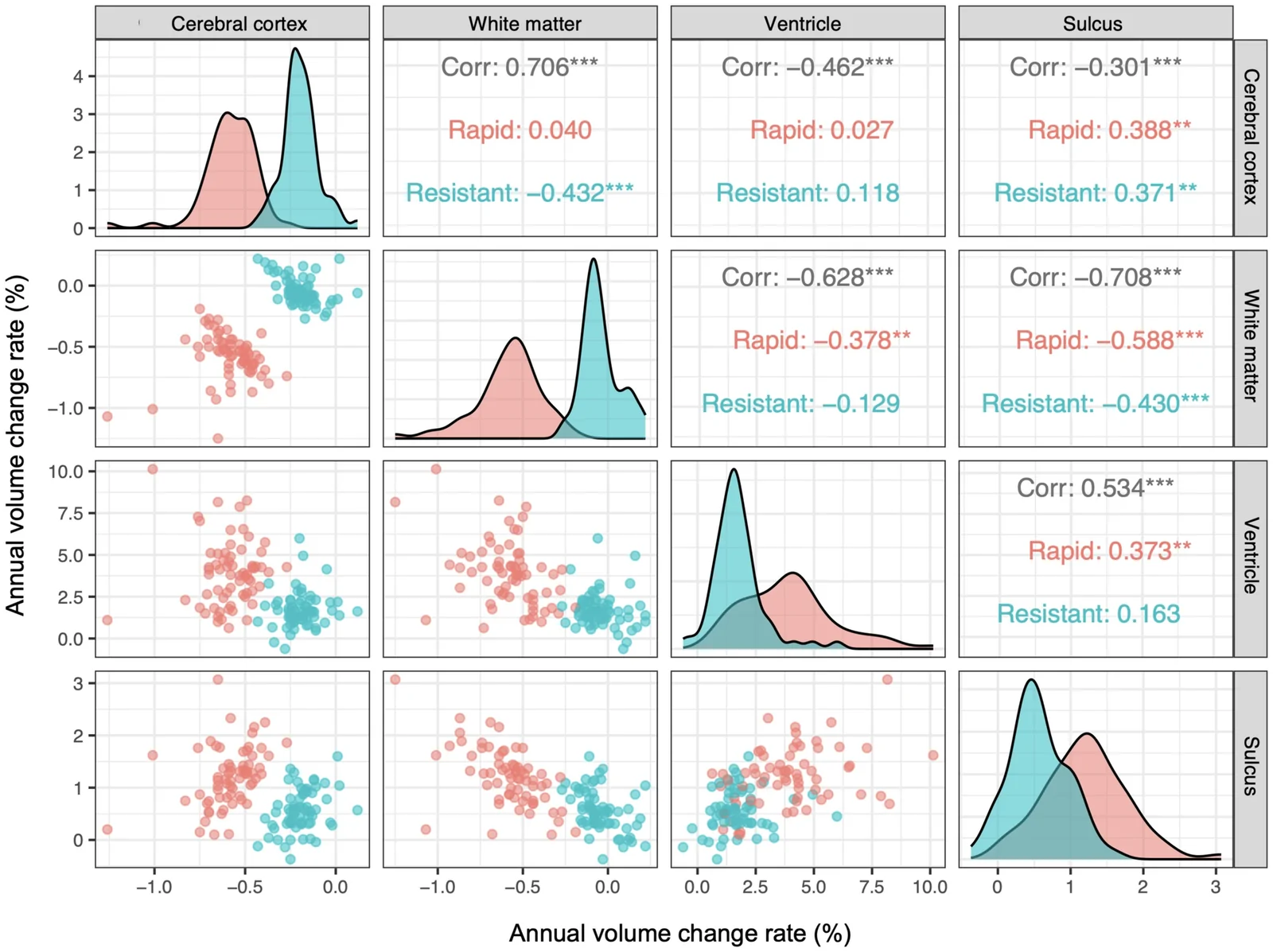
**Brain volume change patterns between rapid and atrophy-resistant groups**. Pairwise scatterplots and density distributions of annual volume change rates (%) are shown. Each dot represents an individual, colour-coded by group (*n* = 66 participants per group). The upper triangle displays group-wise correlation coefficients (Corr), with significance levels indicated (***P < 0.001, **P < 0.01). Statistical comparisons were performed using Pearson correlation for each group for each panel.

To identify distinct patterns of regional brain atrophy, subject-specific annual volume change rates were visualized for individuals classified into the rapid-atrophy and atrophy-resistant groups based on whole-brain atrophy rates (Fig. 6). Rank-based analyses showed that whole-brain atrophy extremes were most closely aligned with cortical atrophy ranks (Supplementary Figs. 2 and 3). Among participants in the top 10% for whole-brain atrophy, the dominant tissue-specific pattern was mixed in 50%, cortex-dominant in 25.8%, white matter-dominant in 15.2% and ventricle-dominant in 1.5%, while 7.6% showed no overlapping compartment-level extreme. Among participants in the bottom 10% for whole-brain atrophy, the pattern was mixed in 40.9%, cortex-preserved in 25.8%, white matter-preserved in 15.2% and low ventricle expansion in 3%, while 15.2% showed no overlapping compartment-level extreme. These findings support the presence of heterogeneous tissue-specific atrophy patterns among individuals at the extremes of whole-brain atrophy.

### Associations with lifestyle and clinical factors

To investigate potential modifiable drivers of whole-brain atrophy, we fitted a fully adjusted linear mixed-effects model and plotted the fixed-effect coefficients for each clinical factor (Supplementary Fig. 4). High BMI [*β* = 0.027; 95% confidence interval (CI) (0.0058–0.049)], elevated diastolic blood pressure (*β* = 0.044; 95% CI 0.021–0.067), smoking exposure (*β* = 0.032; 95% CI 0.012–0.053) and raised LDL-cholesterol (*β* = 0.043; 95% CI 0.021–0.065) were each independently associated with a higher annual whole-brain atrophy rate. In contrast, high glucose status (*β* = 0.0075; 95% CI −0.050 to 0.065), low HDL-cholesterol (*β* = 0.0053; 95% CI −0.049 to 0.060) and high systolic blood pressure (*β* = −0.023; 95% CI −0.072 to 0.026) showed no significant association, suggesting limited independent influence within this cohort.

In the models with each factor on its original continuous scale (Supplementary Fig. 5), the direction of the main associations was consistent with the threshold-based results, with BMI (*β* = 0.0096; 95% CI 0.0060–0.013), diastolic blood pressure (*β* = 0.0033; 95% CI 0.0016–0.0049) and LDL-cholesterol (*β* = 0.0011; 95% CI 0.00052–0.0016) remaining significantly associated with accelerated atrophy, and systolic blood pressure and glucose remaining non-significant. Two factors diverged: smoking was non-significant on its continuous scale (*β* = 0.00044; 95% CI −0.00032 to 0.0012), whereas HDL-cholesterol became significant, with higher levels corresponding to a lower atrophy rate (*β* = −0.0021; 95% CI −0.0028 to −0.0014).

## Discussion

In this long-term longitudinal study of adults with normal cognition, we characterized individuals who had minimal structural atrophy over more than a decade and individuals who showed markedly accelerated decline in brain volume beginning as early as midlife. The atrophy-resistant group in their 70s showed rates as low as 0.27%/year, which was comparable to the population rates in the 40s. Similarly, ventricular enlargement rates ranged from 3.68%/year in the 40s (rapid group) to 1.21%/year in the 70s (resistant group). The average annual rate of ventricular enlargement in the rapid group reached 5.10%/year in individuals in their 50s, which was comparable to the rate observed in Alzheimer’s disease populations.^8,26,27^ Descriptive clustering revealed presence of atrophy phenotypes even among cognitively normal adults. These findings highlight the substantial heterogeneity in age-related brain changes, even among clinically healthy individuals.

Identifying both resilient and vulnerable individuals in a cognitively normal population has implications for early risk stratification and prevention.^28^ From a preventive health perspective, the presence of atrophy-resistant individuals suggests that resilience against structural decline is possible and may be influenced by modifiable lifestyle factors.^12^ In our cohort, non-obesity, lower diastolic blood pressure, minimal smoking exposure and reduced LDL-cholesterol levels were each associated with resistance to brain atrophy, which is consistent with prior studies linking vascular health and brain preservation.^12,29,30^ This study also demonstrates the utility of longitudinal MRI-based stratification of individuals with normal cognition, enabling identification of atypical ageing trajectories. Such phenotypic stratification may guide future research aimed at developing individualized risk assessments and preventive interventions.

We modelled each lifestyle factor both dichotomized at clinical thresholds and on its continuous scale (Supplementary Figs. 4 and 5). Most factors showed consistent directions across both parameterizations, supporting the robustness of the core associations. Divergence emerged for HDL-cholesterol, which was associated with atrophy only when modelled continuously, and smoking, which was associated only when dichotomized; we attribute these to each variable’s measurement properties rather than to conflicting effects. For HDL-cholesterol, the clinical cut-point collapses a continuous gradient into a coarse binary, and as a precisely measured biomarker, it retains its dose–response signal only under continuous coding. Smoking shows the reverse pattern: self-reported pack-years are subject to recall bias and under-reporting that bias estimates towards the null, whereas the ever-versus-never distinction is ascertained more reliably, so the reliability gain outweighed the information lost through dichotomization.

This study did not include amyloid PET, tau PET, or cerebrospinal fluid biomarkers, and the presence of individuals in a preclinical stage of neurodegenerative disease cannot be ruled out.^31^ Moreover, the APOE ε4 genotype, a well-established genetic risk factor for Alzheimer’s disease,^32^ has been linked to accelerated brain atrophy even among individuals with normal cognition.^33^ At the same time, our findings emphasize the heterogeneity of brain ageing, as not all individuals with rapid structural decline progress to cognitive impairment. A subset of adults with normal cognitive ability exhibit atrophy rates similar to those of patients with Alzheimer’s disease but remain symptom-free for over a decade. This supports the theory that neurodegenerative risk and clinical expression are not equivalent and may be modulated by cognitive reserve, resilience factors, or compensatory mechanisms.

Baseline-normalized trajectories address a clinical question that is distinct from absolute volumetry. By normalizing each brain volume to the individual’s baseline scan, our approach focuses on within-person change over time, making it complementary to, rather than a replacement for, cross-sectional normative reference charts.^2^ For example, an individual whose brain volume falls below normative expectations may show little subsequent decline, whereas another whose volume is within the normal range at baseline may exhibit rapid longitudinal atrophy.^9^ We emphasize that the present work is not intended for direct clinical application. Estimating reliable slopes requires multiple imaging time points,^9^ which limits feasibility in routine clinical practice and precludes application to a single cross-sectional scan.

Several limitations should be acknowledged. First, all participants had normal cognition by standard clinical criteria,^15,16^ but biomarkers for neurodegenerative disease, such as amyloid or tau PET,^34^ were not assessed. Consequently, we cannot determine whether some individuals in the rapid-atrophy group had preclinical stages of dementia. In contrast, the presence of atrophy-resistant individuals, including those in their 70s who showed virtually no measurable structural decline over more than a decade, represents a notable finding that cannot be attributed to biomarker status or subclinical pathology. It is also important to note that the acquisition of over 15 years of annual brain imaging would not have been feasible using invasive techniques such as lumbar puncture or PET imaging.^35^ Second, these findings are observational and do not establish causality,^36^ although we identified associations between atrophy phenotypes and lifestyle factors such as blood pressure and smoking exposure. The observed associations between vascular risk factors and brain atrophy may be partially explained by their mediating effects through white matter lesions. Investigating this potential mediation pathway would be an important direction for future research. Building on the findings of our study, future studies should aim to integrate multimodal assessments (e.g. non-imaging biomarkers and genetics) not across entire healthy populations but selectively within stratified subgroups showing atypical trajectories. Such an approach would be both resource-efficient and biologically informative, enabling more detailed understanding of mechanisms underlying resilience and risk. Third, lifestyle and vascular risk factors were associated with atrophy trajectories in our data, but these associations are observational and cannot establish causality; we therefore frame them as candidate risk-enrichment variables rather than validated intervention targets. Establishing causality will require designs beyond observational association, such as randomized interventions with serial MRI slopes as intermediate outcomes. The phenotype introduced in our study could help guide the design of such trials. We regard this risk stratification and trial enrichment value, rather than direct clinical intervention at this stage, as the primary translational contribution of our findings. Ultimately, identifying individuals at elevated or reduced risk within a population with normal cognition has notable implications for personalized prevention and early intervention.^37^

## Conclusion

Overall, this 15-year longitudinal MRI study of adults with normal cognition revealed that structural brain ageing follows highly variable trajectories, ranging from marked atrophy to near-complete resistance. The identification of atypical ageing trajectories in a cognitively normal population through non-invasive imaging highlights the value of longitudinal phenotypic stratification. Future studies should build on this work to develop risk-based monitoring and preventive strategies tailored to individual ageing profiles.

## Supplementary Material

fcag343_Supplementary_Data

## Data Availability

MRI data and individual measurements are currently unavailable because the hospital has withheld them to protect participant privacy. The scripts used for analyses are available via code repository link: https://doi.org/10.5281/zenodo.20819668.

## References

[1] Tenchov R, Sasso JM, Wang X, Zhou QA. Aging hallmarks and progression and age-related diseases: A landscape view of research advancement. ACS Chem Neurosci. 2024;15(1):1–30. 10.1021/acschemneuro.3c00531 PMC10767750 38095562

[2] Bethlehem RAI, Seidlitz J, White SR, et al Brain charts for the human lifespan. Nature. 2022;604(7906):525–533. 10.1038/s41586-022-04554-y PMC9021021 35388223

[3] Fox NC, Schott JM. Imaging cerebral atrophy: Normal ageing to Alzheimer’s disease. Lancet. 2004;363(9406):392–394. 10.1016/S0140-6736(04)15441-X 15074306

[4] Nestor SM, Rupsingh R, Borrie M, et al Ventricular enlargement as a possible measure of Alzheimer’s disease progression validated using the Alzheimer’s disease neuroimaging initiative database. Brain. 2008;131(Pt 9):2443–2454. 10.1093/brain/awn146 PMC2724905 18669512

[5] Scahill RI, Schott JM, Stevens JM, Rossor MN, Fox NC. Mapping the evolution of regional atrophy in Alzheimer’s disease: Unbiased analysis of fluid-registered serial MRI. Proc Natl Acad Sci U S A. 2002;99(7):4703–4707. 10.1073/pnas.052587399 PMC123711 11930016

[6] Chan D, Janssen JC, Whitwell JL, et al Change in rates of cerebral atrophy over time in early-onset Alzheimer’s disease: Longitudinal MRI study. Lancet. 2003;362(9390):1121–1122. 10.1016/S0140-6736(03)14469-8 14550701

[7] Leung KK, Bartlett JW, Barnes J, et al Cerebral atrophy in mild cognitive impairment and Alzheimer disease: Rates and acceleration. Neurology. 2013;80(7):648–654. 10.1212/WNL.0b013e318281ccd3 PMC3590059 23303849

[8] Jack CR Jr, Shiung MM, Gunter JL, et al Comparison of different MRI brain atrophy rate measures with clinical disease progression in AD. Neurology. 2004;62(4):591–600. 10.1212/01.wnl.0000110315.26026.ef PMC2730165 14981176

[9] Di Biase MA, Tian YE, Bethlehem RAI, et al Mapping human brain charts cross-sectionally and longitudinally. Proc Natl Acad Sci U S A. 2023;120(20):e2216798120. 10.1073/pnas.2216798120 PMC10193972 37155868

[10] Fujita S, Mori S, Onda K, et al Characterization of brain volume changes in aging individuals with normal cognition using serial magnetic resonance imaging. JAMA Netw Open. 2023;6(6):e2318153. 10.1001/jamanetworkopen.2023.18153 PMC10308250 37378985

[11] Lindenberger U, von Oertzen T, Ghisletta P, Hertzog C. Cross-sectional age variance extraction: What’s change got to do with it? Psychol Aging. 2011;26(1):34–47. 10.1037/a0020525 21417539

[12] Jia J, Zhao T, Liu Z, et al Association between healthy lifestyle and memory decline in older adults: 10 year, population based, prospective cohort study. BMJ. 2023;380:e072691. 10.1136/bmj-2022-072691 PMC9872850 36696990

[13] Garo-Pascual M, Zhang L, Valentí-Soler M, Strange BA. Superagers resist typical age-related white matter structural changes. J Neurosci. 2024;44(25):e2059232024. 10.1523/JNEUROSCI.2059-23.2024 PMC11209667 38684365

[14] Garo-Pascual M, Gaser C, Zhang L, Tohka J, Medina M, Strange BA. Brain structure and phenotypic profile of superagers compared with age-matched older adults: A longitudinal analysis from the Vallecas project. Lancet Healthy Longev. 2023;4(8):e374–e385. 10.1016/S2666-7568(23)00079-X PMC10397152 37454673

[15] Arevalo-Rodriguez I, Smailagic N, Roqué I Figuls M, et al Mini-Mental State Examination (MMSE) for the detection of Alzheimer’s disease and other dementias in people with mild cognitive impairment (MCI). Cochrane Database Syst Rev. 2015;0(3):CD010783. 10.1002/14651858.CD010783.pub2 PMC6464748 25740785

[16] Elwood RW . The Wechsler Memory Scale-Revised: Psychometric characteristics and clinical application. Neuropsychol Rev. 1991;2(2):179–201. 10.1007/BF01109053 1844708

[17] Elster AD . Gradient-echo MR imaging: Techniques and acronyms. Radiology. 1993;186(1):1–8. 10.1148/radiology.186.1.8416546 8416546

[18] Mugler JP 3rd, Brookeman JR. Rapid three-dimensional T1-weighted MR imaging with the MP-RAGE sequence. J Magn Reson Imaging. 1991;1(5):561–567. 10.1002/jmri.1880010509 1790381

[19] Mori S, Wu D, Ceritoglu C, et al MRICloud: Delivering high-throughput MRI neuroinformatics as cloud-based software as a service. Comput Sci Eng. 2016;18(5):21–35.

[20] Rezende TJR, Campos BM, Hsu J, et al Test-retest reproducibility of a multi-atlas automated segmentation tool on multimodality brain MRI. Brain Behav. 2019;9(10):e01363. 10.1002/brb3.1363 PMC6790328 31483562

[21] Beer JC, Tustison NJ, Cook PA, et al Longitudinal ComBat: A method for harmonizing longitudinal multi-scanner imaging data. Neuroimage. 2020;220(117129):117129. 10.1016/j.neuroimage.2020.117129 PMC7605103 32640273

[22] Fortin JP, Cullen N, Sheline YI, et al Harmonization of cortical thickness measurements across scanners and sites. Neuroimage. 2018;167:104–120. 10.1016/j.neuroimage.2017.11.024 PMC5845848 29155184

[23] Wickham H, Averick M, Bryan J, et al Welcome to the tidyverse. J Open Source Softw. 2019;4(43):1686.

[24] Bates D, Mächler M, Bolker B, Walker S. Fitting linear mixed-effects models using lme4. J Stat Soft. 2015;67:1–48. 10.18637/jss.v067.i01

[25] Bolker B, Robinson D. broom.mixed: Tidying methods for mixed models. R package version 0.2.9.6. 2024. doi:10.32614/cran.package.broom.mixed

[26] Fox NC, Freeborough PA. Brain atrophy progression measured from registered serial MRI: Validation and application to Alzheimer’s disease. J Magn Reson Imaging. 1997;7(6):1069–1075. 10.1002/jmri.1880070620 9400851

[27] Ridha BH, Barnes J, Bartlett JW, et al Tracking atrophy progression in familial Alzheimer’s disease: A serial MRI study. Lancet Neurol. 2006;5(10):828–834. 10.1016/S1474-4422(06)70550-6 16987729

[28] National Academies of Sciences, Engineering, and Medicine, Health and Medicine Division, Board on Health Sciences Policy, Committee on Preventing Dementia and Cognitive Impairment . In: Downey A, Stroud C, Landis S, Leshner AI, eds. Preventing cognitive decline and dementia. National Academies Press; 2017. 28650595

[29] Knopman DS, Penman AD, Catellier DJ, et al Vascular risk factors and longitudinal changes on brain MRI: The ARIC study. Neurology. 2011;76(22):1879–1885. 10.1212/WNL.0b013e31821d753f PMC3115812 21543737

[30] Rabin JS, Pruzin J, Scott M, et al Association of *β*-amyloid and vascular risk on longitudinal patterns of brain atrophy. Neurology. 2022;99(3):e270–e280. 10.1212/WNL.0000000000200551 PMC9302937 35473760

[31] Bateman RJ, Xiong C, Benzinger TLS, et al Clinical and biomarker changes in dominantly inherited Alzheimer’s disease. N Engl J Med. 2012;367(9):795–804. 10.1056/NEJMoa1202753 PMC3474597 22784036

[32] Insel PS, Hansson O, Mattsson-Carlgren N. Association between apolipoprotein E ε2 vs ε4, age, and *β*-amyloid in adults without cognitive impairment. JAMA Neurol. 2021;78(2):229–235. 10.1001/jamaneurol.2020.3780 PMC7551211 33044487

[33] Jansen WJ, Ossenkoppele R, Knol DL, et al Prevalence of cerebral amyloid pathology in persons without dementia: A meta-analysis. JAMA. 2015;313(19):1924–1938. 10.1001/jama.2015.4668 PMC4486209 25988462

[34] Leuzy A, Chiotis K, Lemoine L, et al Tau PET imaging in neurodegenerative tauopathies-Still a challenge. Mol Psychiatry. 2019;24(8):1112–1134. 10.1038/s41380-018-0342-8 PMC6756230 30635637

[35] Evans RW . Complications of lumbar puncture. In: Evans RW, ed. Neurology and trauma. Oxford University Press; 2006:697–715.

[36] Rohrer JM . Thinking clearly about correlations and causation: Graphical causal models for observational data. Adv Methods Pract Psychol Sci. 2018;1(1):27–42.

[37] Yaffe K, Vittinghoff E, Dublin S, et al Effect of personalized risk-reduction strategies on cognition and dementia risk profile among older adults: The SMARRT randomized clinical trial. JAMA Intern Med. 2024;184(1):54–62. 10.1001/jamainternmed.2023.6279 PMC10682943 38010725

