# Supplementary material for: Long-term brain volume trajectories and lifestyle associations in cognitively normal adults: the BRAIN-STRIDE study
Source: Brain Commun. 2026 Sep 7;8(5):fcag343. doi: 10.1093/braincomms/fcag343 (PMC13590224; doi:10.1093/braincomms/fcag343)
Supplement: Supplementary file 1 [file fcag343_supplementary_data.docx]

**Supplementary Online Material**

Fujita S, Mori S, Onda K, et al. Long-term brain volume trajectories and lifestyle associations in cognitively normal adults: the Brain-Stride study

**Supplementary Table 1.** Batch-effect metrics before and after longitudinal ComBat harmonization

**Supplementary Table 2.** Decade-specific annual whole-brain volume change with different thresholds

**Supplementary Figure 1.** Residual volume distributions by scanner before and after longitudinal ComBat harmonization

**Supplementary Figure 2.** Tissue-specific atrophy percentile profiles of participants with rapid and atrophy-resistant whole-brain phenotypes.

**Supplementary Figure 3.** Dominant tissue-specific atrophy patterns among participants at the extremes of whole-brain atrophy

**Supplementary Figure 4.** Associations between clinically defined modifiable health factors and longitudinal whole-brain volume change

**Supplementary Figure 5.** Associations between continuous modifiable health factors and longitudinal whole-brain volume change

**Supplementary Table 1.** Batch-effect metrics before and after longitudinal ComBat harmonization

| **Structure** | **% Variance by Batch** | | **ICC** | | **Kenward–Roger F-test** | |
| --- | --- | --- | --- | --- | --- | --- |
|  | **Before** | **After** | **Before** | **After** | **Before** | **After** |
| Whole brain | 11.1% | 0% | 0.98 | 0.98 | *F*(2, 7322.6) = 2139.22; *p* < 2 × 10^−16^ | *F*(2, 7333.2) = 0.19; *p* = 0.829 |
| Cerebral cortex | 9.0% | 0% | 0.97 | 0.97 | *F*(2, 7366.5) = 1583.44; *p* < 2 × 10^−16^ | *F*(2, 7379.5) = 0.03; *p* = 0.972 |
| White matter | 11.2% | 0% | 0.98 | 0.98 | *F*(2, 7346.6) = 1509.83; *p* < 2 × 10^−16^ | *F*(2, 7357.0) = 0.62; *p* = 0.536 |
| Ventricle | 0% | 0% | 0.96 | 0.95 | *F*(2, 7438.9) = 1.86; *p* = 0.156 | *F*(2, 7457.0) = 0.00; *p* = 0.999 |
| Sulcus | 7.6% | 0.1% | 0.93 | 0.92 | *F*(2, 7522.4) = 627.04; *p* < 2 × 10^−16^ | *F*(2, 7538.6) = 0.07; *p* = 0.930 |
| Frontal lobe | 8.5% | 0% | 0.96 | 0.96 | *F*(2, 7432.6) = 734.60; *p* < 2 × 10^−16^ | *F*(2, 7448.2) = 0.05; *p* = 0.956 |
| Occipital lobe | 7.9% | 0% | 0.93 | 0.93 | *F*(2, 7515.6) = 458.64; *p* < 2 × 10^−16^ | *F*(2, 7532.0) = 0.00; *p* = 0.998 |
| Parietal lobe | 7.9% | 0% | 0.94 | 0.94 | *F*(2, 7503.9) = 1861.85; *p* < 2 × 10^−16^ | *F*(2, 7520.3) = 0.02; *p* = 0.981 |
| Temporal lobe | 8.2% | 0% | 0.95 | 0.95 | *F*(2, 7461.2) = 734.80; *p* < 2 × 10^−16^ | *F*(2, 7479.6) = 0.01; *p* = 0.995 |
| Hippocampus | 14.9% | 0% | 0.90 | 0.90 | *F*(2, 7591.8) = 1354.81; *p* < 2 × 10^−16^ | *F*(2, 7606.3) = 0.00; *p* = 0.999 |

Note.— The overall additive batch (scanner) effect was evaluated by comparing linear mixed-effects models with and without batch as a fixed effect using a Kenward–Roger F-test. Results are reported as F statistics with numerator and denominator degrees of freedom and corresponding p values. No post-hoc pairwise comparisons were performed because the analysis evaluated the overall batch effect rather than differences between individual scanners. A non-significant result after harmonization indicates no statistical evidence of a residual scanner effect. ICC = intraclass correlation coefficient.

**Supplementary Table 2.** Decade-specific annual whole-brain volume change with different thresholds

| Threshold | Decade of baseline age | Typical group  Annual % (IQR) | Rapid atrophy group  Annual % (IQR) | Atrophy-resistant group  Annual % (IQR) |
| --- | --- | --- | --- | --- |
| 5% | 40 s | −0.27 (−0.35 to −0.22) | −0.48 (−0.49 to −0.47) | −0.09 (−0.11 to −0.07) |
|  | 50 s | −0.33 (−0.41 to −0.27) | −0.58 (−0.61 to −0.53) | −0.12 (−0.13 to −0.09) |
|  | 60 s | −0.39 (−0.46 to −0.33) | −0.61 (−0.62 to −0.59) | −0.19 (−0.20 to −0.17) |
|  | 70 s | −0.43 (−0.50 to −0.35) | −1.03 (−1.03 to −1.03) | −0.29 (−0.29 to −0.29) |
| 10% | 40 s | −0.27 (−0.34 to −0.22) | −0.46 (−0.48 to −0.44) | −0.11 (−0.13 to −0.08) |
|  | 50 s | −0.33 (−0.40 to −0.28) | −0.52 (−0.53 to −0.51) | −0.15 (−0.18 to −0.12) |
|  | 60 s | −0.40 (−0.46 to −0.35) | −0.58 (−0.62 to −0.55) | −0.21 (−0.23 to −0.19) |
|  | 70 s | −0.44 (−0.50 to −0.35) | −0.76 (−0.93 to −0.74) | −0.27 (−0.29 to −0.24) |
| 15% | 40 s | −0.27 (−0.34 to −0.23) | −0.44 (−0.47 to −0.42) | −0.13 (−0.15 to −0.11) |
|  | 50 s | −0.33 (−0.39 to −0.29) | −0.52 (−0.53 to −0.49) | −0.16 (−0.19 to −0.14) |
|  | 60 s | −0.39 (−0.44 to −0.35) | −0.55 (−0.59 to −0.53) | −0.23 (−0.24 to −0.20) |
|  | 70 s | −0.43 (−0.48 to −0.37) | −0.78 (−0.98 to −0.75) | −0.31 (−0.31 to −0.29) |

**Supplementary Figure 1.** Residual volume distributions by scanner before and after longitudinal ComBat harmonization

Scanner batch effects before and after longitudinal ComBat harmonization. For every region, the left panel shows data before harmonisation and the right panel after harmonization. After ComBat the coloured box-plots overlap, indicating effective removal of scanner-specific bias. Batch effects were tested across scanners using the Kenward-Roger approximation for fixed effects in a linear mixed-effects model; before harmonization batch was a significant source of variance in nearly all regions (P < 1 × 10⁻¹⁶ for most), the ventricle being the sole exception (P = 0.156). After harmonization, batch-related variance fell to ≤ 0.1% in every region and the batch term was non-significant throughout (P = 0.536–0.999). Residuals were derived from linear mixed-effects models adjusted for age and gender, with a participant-specific random intercept. Boxes show the median and IQR, whiskers extend to 1.5 × IQR, and red markers indicate group means. Open circles indicate individual MRI examinations with residuals beyond the boxplot whiskers. N = 7,915 exams (Signa EXCITE, 360; Discovery MR750, 5,598; Biograph mMR, 1,957).

**Supplementary Figure 2.** Tissue-specific atrophy percentile profiles of participants with rapid and atrophy-resistant whole-brain phenotypes

Each row represents one participant, ordered by whole-brain atrophy, and each column shows that participant's percentile rank for whole brain, cortex, white matter, ventricle, and sulcal measures. Color indicates percentile (dark red = high atrophy/100th percentile; dark blue = low atrophy/0th percentile). The top panel shows participants with a rapid atrophy phenotype (top 10% for whole-brain atrophy) and the bottom panel shows those with an atrophy-resistant phenotype (bottom 10% for whole-brain atrophy). While whole-brain ranks are uniformly extreme by design, the tissue-specific columns show substantial within-individual heterogeneity, indicating that whole-brain atrophy extremes do not correspond to uniform extremes across all tissue compartments. N = 66 participants for each group.

**Supplementary Figure 3.** Dominant tissue-specific atrophy patterns among participants at the extremes of whole-brain atrophy

Dominant tissue-specific atrophy patterns among participants at the extremes of whole-brain atrophy, defined by overlap with compartment-level top or bottom 10%. Bars show the number and percentage of participants in each dominant pattern category. GM, gray matter; WM, white matter. N = 653 participants.

**Supplementary Figure 4.** Associations between clinically defined modifiable health factors and longitudinal whole-brain volume change

Forest plot showing fixed-effect estimates for the interaction between each dichotomized clinical risk factor and follow-up time. Each binary flag contrasts participants who crossed a clinical risk threshold with those who did not. Estimates are plotted relative to zero (vertical dashed line); negative values indicate relative preservation of brain volume (atrophy-resistant), whereas positive values indicate greater volume loss (atrophy-prone). Statistical significance of each time-by-risk-factor interaction was evaluated using a two-sided t-test. The results were as follows: BMI, t(7208) = 2.73, p = 0.006, pBH = 0.011; glucose, t(7208) = 0.42, p = 0.675, pBH = 0.675; smoking, t(7208) = 3.34, p < 0.001, pBH = 0.003; HDL, t(7208) = 0.81, p = 0.416, pBH = 0.486; LDL, t(7208) = 3.18, p = 0.001, pBH = 0.003; sBP, t(7208) = −1.19, p = 0.235, pBH = 0.329; and dBP, t(7208) = 3.22, p = 0.001, pBH = 0.003. pBH values were adjusted across the seven tests using the Benjamini–Hochberg procedure. No post-hoc comparisons were performed because each model included a single binary contrast. N = 653 participants. BMI, body mass index; HDL, high-density lipoprotein cholesterol; LDL, low-density lipoprotein cholesterol; sBP, systolic blood pressure; dBP, diastolic blood pressure.

**Supplementary Figure 5.** Associations between continuous modifiable health factors and longitudinal whole-brain volume change

Forest plot showing fixed-effect estimates for the interaction between each continuous clinical factor and follow-up time. For slope analyses, β coefficients for the time-by-factor interaction represent the difference in annual whole-brain volume change per one-unit increase in each factor. Estimates are plotted relative to zero (vertical dashed line); negative values indicate relative preservation of brain volume (atrophy-resistant), whereas positive values indicate greater volume loss (atrophy-prone). Statistical significance of each time-by-factor interaction was evaluated using a two-sided t-test. The results were as follows: BMI, t(7208) = 5.25, p < 0.001, pBH < 0.001; glucose, t(7208) = 0.97, p = 0.331, pBH = 0.331; smoking, t(7208) = 1.13, p = 0.256, pBH = 0.299; HDL, t(7208) = −5.69, p < 0.001, pBH < 0.001; LDL, t(7208) = 3.89, p < 0.001, pBH < 0.001; sBP, t(7208) = −1.57, p = 0.116, pBH = 0.162; and dBP, t(7208) = 3.92, p < 0.001, pBH < 0.001. pBH values were adjusted across the seven tests using the Benjamini–Hochberg procedure. Post-hoc comparisons were not applicable because the predictors were analyzed as continuous variables. N = 653 participants. BMI, body mass index; HDL, high-density lipoprotein cholesterol; LDL, low-density lipoprotein cholesterol; sBP, systolic blood pressure; dBP, diastolic blood pressure.
